# Rapid Assessment of Soil Headspace Dynamics Under Denitrification-Promoting Conditions via an IoT-MOX Sensor Array

**DOI:** 10.3390/s26216768

**Published:** 2026-10-26

**Authors:** Elisabetta Poeta, Giulia Daly, Agnese Bellabarba, Estefanía Núñez-Carmona, Alessandra Adessi, Filippo Rocchi, Dario Belmonte, Veronica Sberveglieri

**Affiliations:** 1 Institute of Bioscience and Bioresources (CNR-IBBR), National Research Council, URT-Reggio Emilia, Via J.F. Kennedy, 17/i, 42124 Reggio Emilia, Italy; elisabettapoeta@cnr.it (E.P.); veronica.sberveglieri@cnr.it (V.S.); 2 Department of Agricultural, Food, Environmental and Forestry Sciences (DAGRI), University of Florence, 50144 Firenze, Italy; dalygiulia@gmail.com (G.D.); agnese.bellabarba@unifi.it (A.B.); alessandra.adessi@unifi.it (A.A.); 3 Genexpress Laboratory, Department of Agronomy, Food, Environmental and Forestry Sciences (DAGRI), University of Florence, 50019 Sesto Fiorentino, Italy; 4 Research Centre for Agriculture and Environment, Consiglio per la Ricerca in Agricoltura e l’Analisi dell’Economia Agraria (CREA-AA), 50125 Florence, Italy; filippo.rocchi@crea.gov.it; 5 Department of Biological, Chemical and Pharmaceutical Sciences and Technologies (STEBICEF), University of Palermo, Viale delle Scienze, Ed. 16, 90128 Palermo, Italy; dario.belmonte@unipa.it; 6 Nano Sensor Systems SRL (NASYS), Via Alfonso Catalani, 9, 42121 Reggio Emilia, Italy

**Keywords:** soil microcosms, denitrification, volatilomics, MOX sensors, electronic nose, gas sensing, multivariate analysis

## Abstract

Real-time monitoring of soil gaseous emissions is important for understanding nitrogen transformations and associated emissions of reactive and greenhouse gases. In this study, controlled soil microcosms were used to investigate changes in gas emissions, volatile profiles, and microbial functional markers under control and denitrification-promoting conditions, and to explore the ability of an IoT-enabled metal oxide (MOX) gas sensor array to monitor dynamic changes in the overall soil headspace through multivariate resistance-response patterns. The experimental workflow included preliminary Fourier transform infrared (FTIR) gas analysis on dedicated microcosms to verify the response induced by the BOOST treatment and identify relevant monitoring windows. Separate microcosms were subsequently investigated by solid-phase microextraction gas chromatography–mass spectrometry (SPME–GC–MS), qPCR targeting key nitrogen-cycling functional genes, and MOX-based headspace monitoring. BOOST microcosms, amended with mineral nitrogen and glucose and maintained at 100% field capacity, exhibited an early increase in N_2_O emissions, with the highest measured concentration observed at 24 h, together with changes in bacterial abundance and nitrogen-cycling functional gene profiles. Volatilome analysis revealed a broader distribution of detected VOCs among chemical classes under BOOST conditions, with marked temporal changes and the emergence or enrichment of specific VOC classes, particularly at intermediate sampling times. MOX measurements captured concurrent changes in the overall headspace response and generated distinct resistance-based response patterns across treatments and sampling times. Exploratory multivariate analysis showed separated response clusters, which were interpreted alongside the temporal changes independently observed by FTIR and GC–MS. Overall, the findings indicate that MOX sensor arrays can rapidly capture complex and time-dependent response patterns associated with changes in the overall soil headspace under controlled conditions.

## 1. Introduction

Nitrogen (N) availability is a major determinant of agricultural productivity, although its transformations in soil are governed by complex interactions among microbial communities, physicochemical conditions, and management practices [1,2,3,4]. A substantial fraction of the nitrogen applied to agricultural soils may be lost through leaching or gaseous emissions, reducing nitrogen-use efficiency and contributing to environmental impacts [3]. Among these transformations, denitrification involves the sequential microbial reduction of nitrate and nitrite to nitric oxide (NO), nitrous oxide (N_2_O), and ultimately dinitrogen (N_2_). Although this process limits nitrate accumulation, incomplete denitrification can result in the release of N_2_O, a potent greenhouse gas and a major contributor to stratospheric ozone depletion [5,6].

The relative contributions of nitrification and denitrification to soil N_2_O emissions depend on multiple interacting factors, including soil type, nitrogen and carbon availability, oxygen status, temperature, and water content [7,8,9,10,11,12,13]. Soil moisture is particularly relevant because it regulates gas diffusion and oxygen availability, thereby influencing the balance between aerobic and anaerobic microbial processes. Rapid changes in water content may also generate transient emission peaks that are difficult to capture using discontinuous sampling strategies [14,15]. Recent syntheses have further emphasized the dependence of denitrification rates on local soil properties and environmental conditions, highlighting the need for analytical approaches capable of resolving both temporal dynamics and matrix-related variability [1].

Despite advances in the understanding of soil nitrogen cycling, monitoring its gaseous and metabolic signatures remains analytically challenging. Established techniques such as gas chromatography and Fourier transform infrared spectroscopy provide selective and quantitative information on target gases, including N_2_O, NO, NO_2_, NH_3_, CH_4_, and CO_2_. Rapid mid-infrared laser-spectroscopic approaches have also been applied to high-frequency measurements of N_2_O isotopologues and to the investigation of N_2_O source processes in soil systems [16]. However, these techniques require specialized instrumentation and primarily target individual gaseous species, providing limited information on the overall soil volatile profile. Isotope-based approaches can quantify denitrification and distinguish N_2_ and N_2_O production, but their complexity limits their application to rapid monitoring. Consequently, no single conventional technique simultaneously provides compound-specific gas measurements, comprehensive volatile profiling, and rapid assessment of headspace dynamics.

Soil volatilomics has emerged as a complementary approach to investigating microbial and physicochemical processes through the analysis of volatile organic compounds (VOCs). Previous studies have shown that soil VOC profiles reflect microbial activity, water availability, plant–soil interactions, and agricultural management practices [17,18,19,20]. Headspace solid-phase microextraction coupled with gas chromatography–mass spectrometry (HS-SPME–GC–MS) enables the identification of a broad range of volatile compounds [21], although its offline and time-consuming nature limits its suitability for rapid monitoring.

Gas-sensing technologies encompass a broad range of sensing materials and architectures, including metal oxides, two-dimensional materials, and other chemiresistive platforms developed for the detection of gaseous species across environmental, industrial, and safety-related applications [22].

Metal oxide semiconductor (MOX) sensor arrays provide a rapid and cost-effective approach for monitoring complex gaseous mixtures. Variations in the electrical resistance of differently functionalized sensing elements generate multidimensional response patterns that represent the overall headspace composition rather than individual compounds [23,24,25,26]. Beyond conventional MOX-based platforms, other chemiresistive electronic-nose architectures employing hybrid nanostructured sensing materials have also been investigated for VOC detection and discrimination, including carbon nanotube–TiO_2_ hybrid systems [27]. Owing to their compact design, compatibility with automated acquisition, and potential integration with IoT platforms, MOX-based systems have been increasingly investigated for environmental, biomedical, and agri-food applications, as well as for integration into wearable and IoT-enabled monitoring systems [22,23,24,25,26,27,28]. Their application to complex matrices such as soil nevertheless requires careful consideration of humidity, temperature, sensor drift, and environmental variability. When trained and validated on representative datasets, machine-learning models may help distinguish informative response patterns from interfering environmental effects, thereby improving the robustness of condition-related pattern recognition. Following appropriate biological and field validation, MOX arrays may therefore provide a complementary rapid-screening tool for identifying condition-associated changes in complex headspace profiles.

In the present study, this concept was implemented using a custom SnO_2_-based MOX sensor array developed and fabricated by CNR-IBBR and integrated into the S3+ platform, a dedicated system providing the fluidic handling, signal acquisition, and IoT connectivity required for sensor operation. The platform was combined with complementary reference techniques to characterize the evolution of a dynamic and chemically complex soil headspace.

Targeted gas measurements, soil volatilomics, and sensor arrays provide complementary information on soil processes, differing in chemical specificity and monitoring capabilities. In this study, preliminary FTIR measurements were performed on dedicated soil microcosms to characterize the gaseous response to the BOOST treatment and identify relevant monitoring windows. Separate microcosms were then analyzed using SPME–GC–MS, qPCR, and MOX sensors to investigate VOC profiles, nitrogen-cycling functional markers, and overall headspace responses, respectively.

We hypothesized that changes in nitrogen and carbon availability and soil moisture would induce time-dependent variations in gaseous emissions, VOC composition, and microbial functional markers, potentially reflected in distinct MOX resistance-based response patterns. Accordingly, the study aimed to assess the ability of an IoT-enabled MOX sensor array to capture changes in soil headspace composition under controlled denitrification-promoting conditions, supported by complementary chemical and molecular analyses. This preliminary investigation provides a methodological basis for future validation using biologically independent microcosms and field-relevant conditions.

## 2. Materials and Methods

### 2.1. Setup of Soil Laboratory Microcosms for MOX Sensor Response Characterization

For the setup of soil laboratory-based microcosms, soil was sampled from an experimental area located at Tenuta di Cesa in Marciano della Chiana (Arezzo, Italy—43°18′36.5″ N 11°49′32.8″ E) in July 2023. Soil samples were collected from the top 20 cm of the mineral soil layer using a steel cylinder, then sieved to 2 mm and stored at 4 °C. Then, 200g of homogenized soil was placed in air-tight jars of 1 L volume equipped with two-way valves for headspace gas sampling. To promote denitrification, BOOST microcosms were amended with mineral N (0.40 g N kg^−1^ soil, supplied as KNO_3_) and glucose (1%), as previously tested [29], and were wetted to 100% of field capacity (FC). Mineral N and glucose were dissolved in the distilled water used to reach 100% FC. CONTROL microcosms received no mineral N or glucose and were wetted with distilled water only to 25% of FC.

### 2.2. FTIR Analysis

For the gas measurements with the FTIR Gas Analyzer, three soil microcosms were set up as described in the previous section for both boosted and control treatments. Microcosms were incubated at 25 °C in the dark for 3, 24, 48, and 144 h after wetting. The initial gas emissions were determined by sampling the headspace 3 h after closing the jars (t3). After the second sampling (T24), the jars were ventilated and then resealed for 24 h to allow gas accumulation before the subsequent measurement at T48. Jars were then left open for 120 h and then closed again for 24 h before performing the last sampling at 144 h (t144). At each sampling time, additional empty jars were incubated as blanks to account for gas background concentration. Gas concentrations of N_2_O, NO_2_, NO, NH_3_, CH_4_, and CO_2_ were measured by a DX4040 FTIR Gas Analyzer (Gasmet Technologies Oy, Vantaa, Finland). At each sampling occasion, the jar valves were connected to the gas analyzer using two 6 mm Teflon tubes (inlet and outlet) for measuring the gas concentration in ppm. Differences in gas emissions between BOOST and CONTROL groups were evaluated at each time point using Student’s *t*-tests through the stats (v.4.1.2) and ggpubr (v.0.6.0) packages in RStudio (v.4.4.3). In both tests, the Benjamini–Hochberg correction for multiple comparisons was applied.

### 2.3. Soil Laboratory Microcosms for MOX Sensors Training

Based on the temporal trends identified during the preliminary FTIR experiment, dedicated microcosms were subsequently prepared for MOX, GC–MS, and qPCR analyses using the same homogenized soil batch and the same CONTROL and BOOST treatments described above. These microcosms were incubated at 25 °C in the dark. For the MOX measurements, one microcosm was used for each experimental condition. Repeated acquisitions performed on the same microcosm were considered technical replicates and were used to assess instrumental repeatability and short-term within-sample signal variability. These acquisitions were performed consecutively within the same measurement session, minimizing the time interval between technical replicates. MOX measurements were carried out using a semi-continuous acquisition protocol consisting of consecutive measurement cycles. Each cycle included an initial stabilization phase under clean air, followed by exposure of the sensor array to the microcosm headspace and a final clean-air purging phase to allow signal recovery before the subsequent acquisition. Headspace sampling for GC–MS analysis was performed at 3, 24, 48, 72, and 144 h after wetting. MOX and GC–MS measurements were performed within comparable experimental time windows, although on dedicated microcosms and not as simultaneous measurements. Moreover, soil samples for qPCR analysis were collected immediately after wetting and at 24 and 48 h after opening the jars and then stored at −80 °C until DNA extraction.

### 2.4. qPCR Analysis

Soil DNA was extracted from 0.3 g using the FastDNA Spin Kit for Soil (MP Biomedicals, Solon, OH, USA) in accordance with the manufacturer’s protocol. DNA concentration and purity were verified with a NanoDrop Lite Plus spectrophotometer (Thermo Fisher Scientific, Wilmington, NC, USA). Target gene abundances were quantified through qPCR using an ABI StepOnePlus Real-Time PCR System (Applied Biosystems, Foster City, CA, USA), with Syber GreenER qPCR SuperMix for iCycler (Invitrogen Life Technologies, Milan, Italy), and the primers listed in Supplementary Materials Table S1. The thermal cycling conditions consisted of an initial activation performed at 95 °C for 10 min, followed by 40 cycles of denaturation at 95 °C for 10 s, then a gene-specific annealing as reported in Table S1, and elongation at 72 °C for 10 s. A final melting curve analysis was performed (95 °C for 10 s, 60 °C for 15 s, followed by a ramp to 95 °C at 0.1 °C/s) before a 40 °C cooling step for 30 s. Three technical replicates were performed, including three negative controls, and results were expressed as gene copy numbers per gram of soil. The N_2_O reduction potential was determined as previously reported [30,31]. Briefly, the N_2_O reduction index was calculated as the ratio of *nosZ* gene abundance to the combined abundance of *nirS* and *nirK*, with all gene abundances expressed as gene copy numbers per gram of soil. Conversely, the N_2_O production index was calculated as the inverse ratio. Statistical analyses were performed in RStudio (v.4.4.3) [32]. For all gene abundances and functional indices, significant differences between control and denitrification-boosted soil microcosms were evaluated at each sampling time using Student’s *t*-tests or non-parametric Wilcoxon rank-sum tests via the stats (v.4.1.2) and ggpubr (v.0.6.0) packages. In both tests, the Benjamini–Hochberg correction for multiple comparisons was applied.

### 2.5. S3+ Electronic Nose and Gas Sensor Technology

The experiments were conducted using the S3+ electronic nose platform, developed by Nano Sensor Systems srl (NASYS, Reggio Emilia, Italy), integrating a custom MOX sensor array developed and fabricated by the Institute of Biosciences and Bioresources of the National Research Council of Italy (CNR-IBBR, URT, Reggio Emilia, Italy). The sensing array consists of two independent sensor units, each incorporating three SnO_2_-based sensing elements: undoped SnO_2_, Pd-functionalized SnO_2_, and Au-functionalized SnO_2_, for a total of six active sensors. The CNR-IBBR-developed sensing elements were integrated into the S3+ fluidic and acquisition platform for controlled headspace analysis and IoT-compatible data transmission (Table 1).

All sensing elements are based on tin dioxide (SnO_2_), including both pristine and noble-metal-functionalized variants. Specifically, the array comprises two undoped SnO_2_ sensors, two Pd-functionalized SnO_2_ sensors, and two Au-functionalized SnO_2_ sensors, selected to provide complementary response characteristics toward chemically diverse components of complex gaseous mixtures.

Each sensing element was operated at a constant working temperature of 500 °C, regulated by integrated micro-heaters to ensure stable and reproducible operating conditions. The sensing chamber (11 × 6.5 × 1.3 cm) was designed to promote uniform gas flow across the sensor surfaces, thereby reducing concentration gradients and supporting reproducible responses during repeated acquisition cycles.

### 2.6. Sensor Calibration and Data Acquisition

Prior to experimental measurements, the MOX sensor array underwent a standardized conditioning procedure to stabilize the sensing layers and ensure long-term signal reliability. This procedure included an initial thermal treatment, during which the sensors were exposed to elevated temperatures (500–800 °C) for variable durations (1–10 h), followed by an extended stabilization phase under continuous clean-air flow to equilibrate the baseline electrical resistance [33]. Sensor calibration was subsequently performed using certified reference gases representative of common fermentation-related volatile compounds, including ethanol and acetone. Controlled exposures to known concentrations of these analytes were used to verify sensor responsiveness, technical repeatability, and consistency among sensing elements. Gas analysis was performed using a fixed acquisition protocol at a sampling flow rate of 75 sccm, consisting of three consecutive phases: (i) baseline stabilization under clean air for 300 s, (ii) exposure to the sample headspace for 300 s, and (iii) recovery under clean-air conditions for 100 s. Each acquisition therefore lasted 700 s. For each sample, five repeated measurement cycles were performed on the same microcosm. These cycles were considered technical replicates and were used to assess instrumental repeatability and short-term within-sample signal variability. Electrical resistance values were continuously recorded at a sampling rate of 1 Hz. Sensor responses were expressed as normalized resistance ratios (R/R_0_), where R is the instantaneous resistance and R_0_ corresponds to the average resistance measured during the initial stabilization phase. To enhance signal comparability and reduce high-frequency noise, the resistance time series were baseline-corrected and smoothed using a Savitzky–Golay filtering algorithm prior to exploratory multivariate analysis.

### 2.7. Gas Chromatography Coupled with Mass Spectrometric Detection (GC–MS) Analysis

Volatile organic compound profiles were characterized by solid-phase microextraction coupled with gas chromatography–mass spectrometry (SPME–GC–MS) to provide complementary chemical information for the interpretation of the MOX sensor responses. Dedicated GC–MS microcosms were prepared using the same homogenized soil batch employed for the FTIR and MOX measurements and applying the same CONTROL and BOOST treatments. The microcosms consisted of jars comparable in size to those used for the MOX analysis, but equipped with a dedicated lid containing a suitable sampling port that allowed the SPME fiber to be exposed directly to the headspace without opening or disturbing the sample. GC–MS measurements were therefore performed on separate microcosms specifically prepared for this analytical platform.

Headspace extraction was carried out using a divinylbenzene/carboxen/polydimethylsiloxane (DVB/CAR/PDMS) fiber, selected for its broad affinity toward volatile compounds with different physicochemical properties. The fiber was exposed to the sample headspace at 30 °C for 90 min. After extraction, the analytes were thermally desorbed in the GC injector port in splitless mode for 6 min at 250 °C to maximize their transfer onto the analytical column.

Chromatographic analysis was performed using a Shimadzu GC-2020 gas chromatograph coupled to a Shimadzu QP2020 mass spectrometer (Kyoto, Japan). Separation was achieved on a MEGA-5MS low-polarity capillary column (25 m × 0.25 mm internal diameter × 0.25 μm film thickness), suitable for the analysis of volatile and semi-volatile compounds. Hydrogen of 99.99% purity, generated using a GENius PF500 system (FullTech Instruments Srl, Rome, Italy), was used as the carrier gas at a constant flow rate of 2.5 mL min^−1^. The hydrogen supply was operated under controlled flow and pressure conditions and was used exclusively as a carrier gas.

The GC oven temperature program started at 40 °C, held for 1 min, followed by a ramp of 4.5 °C min^−1^ to 50 °C, a second ramp of 6.5 °C min^−1^ to 80 °C, and a final ramp of 15 °C min^−1^ to 180 °C. The total chromatographic run time was 17 min, while the overall analysis time, including headspace extraction, was approximately 107 min.

The mass spectrometer was operated in electron ionization mode at 70 eV, with the ion source maintained at 240 °C. Mass spectra were acquired in total ion current mode over a mass-to-charge range of 35–500 *m*/*z*, using a scan interval of 0.3 s.

Compound identification was performed by comparing the acquired mass spectra with the NIST11, NIST11b, and FFNSC2 reference libraries. Only compounds showing a spectral similarity score above 90% were retained for further interpretation. Peak integration was based on peak area, using a minimum area threshold of 500 AMU. Data-processing parameters included a slope of 100 min^−1^, a peak width of 2 s, a drift value of 0 min^−1^, and a doubling time of 1000 min. No signal smoothing was applied to preserve the integrity of the raw chromatographic data. Relative VOC abundance was expressed as the percentage contribution of each peak area to the total chromatographic area.

The resulting VOC profiles provided complementary qualitative and semi-quantitative information on the relative composition of the soil headspace under the investigated experimental conditions, supporting the interpretation of the MOX sensor response patterns without implying direct quantitative calibration or compound-specific detection.

### 2.8. Data Analysis and Statistical Processing

Sensor responses were recorded as time-dependent changes in electrical resistance and transmitted to a cloud-based platform for data storage and processing. The raw time-series data from the six sensing elements are provided in Supplementary Materials, Annex S1. Individual sensor profiles, obtained from pure SnO_2_, Pd-doped SnO_2_, and Au-doped SnO_2_ sensing materials, were initially examined to characterize their temporal behavior.

Raw resistance signals were normalized to the initial baseline resistance (R_0_) to improve comparability across sensing elements and measurement cycles. For each acquisition, the variation between the initial resistance and the minimum resistance recorded during headspace exposure was determined. Five measurement cycles were performed on each microcosm and considered technical replicates to evaluate short-term instrumental repeatability. The normalized resistance ratio (R/R_0_) and its standard deviation were calculated across these cycles, and only datasets meeting the predefined maximum relative uncertainty threshold of 10% were retained for further analysis.

Numerical descriptors were subsequently extracted from the normalized time series to characterize the dynamic sensor responses. These included the minimum and maximum first derivatives, area under the response curve, response amplitude (ΔR), signal-to-noise ratio, overall mean, mean of the final 60 recorded values, minimum and maximum values, and difference between the means of the final and initial signal segments. The area under the response curve was calculated using Simpson’s rule.

A Random Forest algorithm was applied to obtain an exploratory ranking of the extracted features according to their association with predefined treatment- and time-related acquisition groups. The highest-ranked features were then used as input variables for Linear Discriminant Analysis (LDA) to visualize multivariate sensor-response patterns across experimental conditions and sampling times. Both analyses were performed on the same dataset and were intended exclusively for exploratory purposes, without estimating predictive performance or classification generalizability.

Because only one independently prepared microcosm was available for each experimental condition, the five repeated acquisitions represented technical rather than biological replicates. Consequently, microcosm-specific variability could not be distinguished from treatment-related effects, and independent biological validation or microcosm-level cross-validation could not be performed. The resulting multivariate patterns were therefore interpreted as descriptive characteristics of the investigated dataset rather than evidence of reproducible treatment discrimination. Further validation using independent biological replicates and more variable environmental conditions will be required to establish the reproducibility and predictive potential of the proposed approach.

## 3. Results and Discussion

### 3.1. Tuning of Soil Laboratory Microcosms for Training of MOX Sensors: Setup and Monitoring of N-Cycling Functional Gene Abundance in Soil Bacterial Communities

Soil laboratory microcosms were established to characterize the MOX sensor response within a controlled, semi-natural environment. Two distinct conditions were simulated to monitor soil nitrogen emissions: a putative denitrification-boosted treatment and an untreated control. A preliminary experiment, using a Gasmet FTIR analyzer, was conducted in an attempt to (i) validate the microcosm setup as a representative proxy for the simulated conditions for denitrification and (ii) determine the optimal monitoring duration for gas emissions. Gas emission dynamics measured via FTIR spectroscopy revealed distinct temporal patterns over the 144 h of incubation between the BOOST and the control treatment (Supplementary Materials Figure S1). The combined BOOST treatment induced significantly marked changes in nitrogen-related gas emissions during the first 24 h (Supplementary Materials Figure S1). In particular, the BOOST treatment significantly increased the N_2_O emission rate at 24 h compared to the control before declining to baseline levels (Supplementary Materials Figure S1A). Our findings aligned with the N_2_O emissions profiles previously observed after the rewetting procedure of degraded soils under laboratory conditions [34]. Conversely, the highest NO_2_ emission rate was detected at 48 h in the BOOST treatment, which maintained significantly elevated rates throughout the monitoring window (Figure S1B). Emissions of NO, NH_3_, and CH_4_ showed initial transient spikes within the first hours in both BOOST and control treatments and subsequently exhibited a progressive decrease, maintaining low emission levels (Figure S1C–E). CO_2_ respiration rates in the BOOST treatment reached the highest emission rate at 48 h, significantly higher than the control, demonstrating a marked increase in overall soil respiration (Figure S1F). Overall, these findings suggested that our microcosm setup can be a semi-natural proxy for concurrently evaluating the N-related microbial guilds’ dynamics during potential soil denitrification in the context of MOX sensor calibration. Soil microcosms provided a controlled environment for sensor calibration, as validated in previous studies for their ability to mimic soil nitrogen transformation kinetics [35]. By maintaining stable conditions and, simultaneously, boosting denitrification through nutrient amendments and recreating anaerobic conditions, our soil microcosms can provide the emission dynamics within a controlled environment for optimizing the response of the MOX sensors under representative, semi-natural conditions.

Quantitative PCR (qPCR) was employed to estimate the abundance of bacterial nitrogen-cycling communities within both control and denitrification-boosted soil microcosms. The target functional genes served as proxies for the functional potential of N-cycle-related microbial guilds: *nifH* was used to quantify nitrogen-fixing populations, while *amoA* (AOB) served as a marker for ammonia-oxidizing bacteria [36]. To characterize the denitrification pathway, the *nirK* and *nirS* genes were targeted, as they encode structurally distinct nitrite reductases responsible for reducing nitrite (NO_2_^−^) to nitric oxide (NO). Additionally, the *nosZ* gene, which encodes the nitrous oxide reductase that facilitates the reduction of nitrous oxide (N_2_O) to dinitrogen gas (N_2_), was quantified to assess the final step of the denitrification process. Total bacterial abundance of soil microcosms for GC-MS analysis was significantly higher in the boosted microcosms at T24 (Supplementary Materials Figure S2A). At T48, the nitrogen-fixing community exhibited a decline in the boosted microcosms compared to the control despite an initial increase at T24 (Supplementary Materials Figure S2B). Similarly, the nitrifying community of control microcosms showed a consistent increase in *amoA* abundance, likely due to the lack of nitrate addition, while the boosted soils maintained significantly lower levels at both T24 and T48 (Supplementary Materials Figure S2F). The lower AOB abundance observed in BOOST soils was consistent with previous studies showing that high and prolonged N inputs may alter ammonia-oxidizing communities and, under specific soil conditions, reduce AOB abundance while increasing N_2_O emissions [37,38,39]. The microbial community dynamics in the MOX sensor microcosms followed a similar successional pattern to those observed in the GC-MS setup for the total bacterial abundance (Supplementary Materials Figure S3A) and for the nitrogen-fixing community (Supplementary Materials Figure S3B). Instead, the nitrifying bacteria (*amoA*) abundance in boosted microcosms was significantly higher at T0 and at T48 (Supplementary Materials Figure S3F).

**Figure 1:**
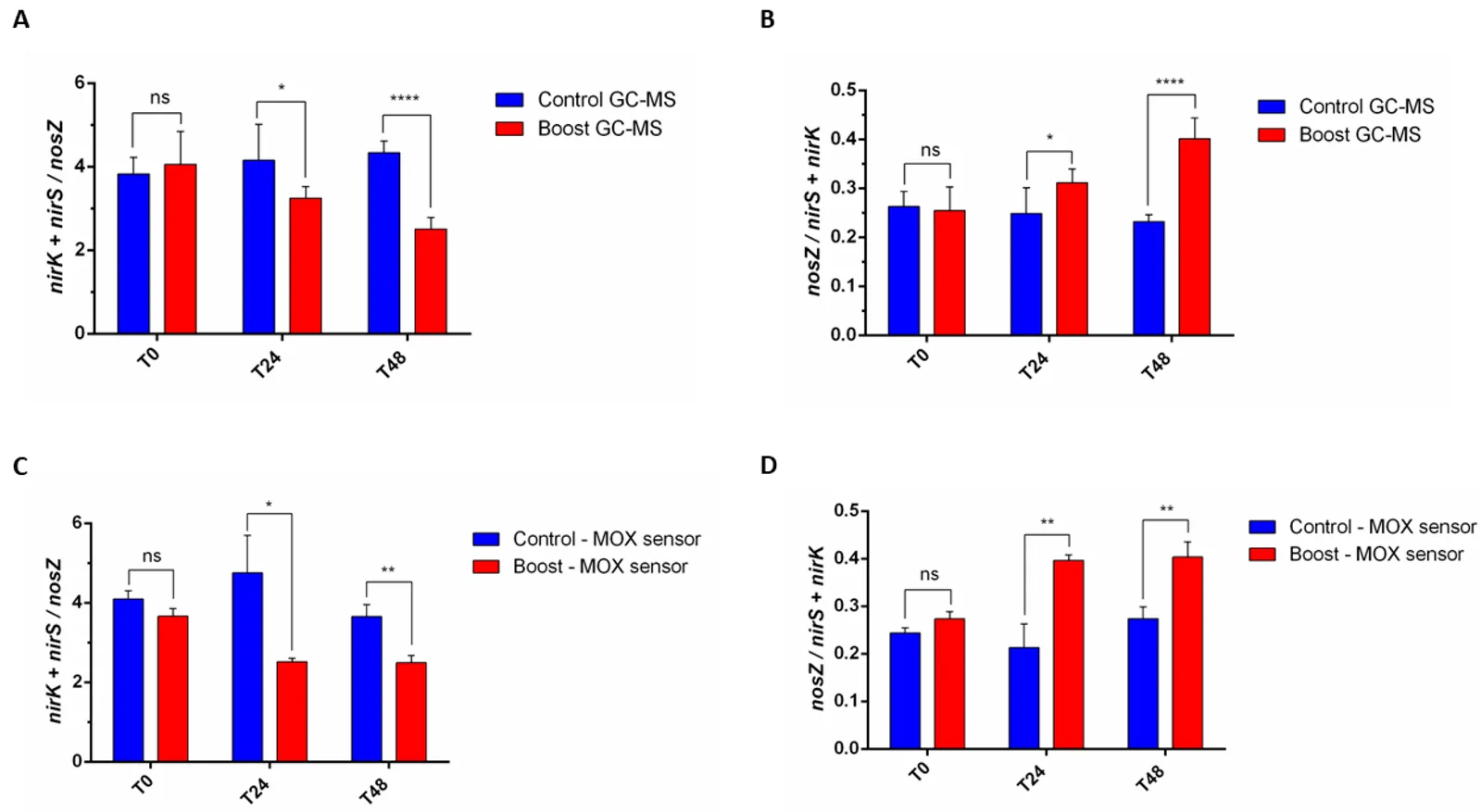
N_2_O production potential (**A**,**C**) and N_2_O reduction potential (**B**,**D**) of soil bacterial communities in control and denitrification-boosted soil laboratory microcosms settled for GC-MS and MOX sensor analysis. The error bars indicate ± the standard errors, whereas the bars represent the mean values. Asterisks in bar plots indicate statistically significant differences (ns, not significant; * *p* < 0.05; ** *p* < 0.01; **** *p* < 0.0001).

To predict the microbial potential of N_2_O emission, the N_2_O reduction and N_2_O production potentials were estimated. The putative denitrification potential of the soil bacterial communities appeared to reflect distinct temporal shifts following the treatment for boosting denitrification (Figure 1). At T0, no significant differences were observed between control and boosted soil microcosms for either the estimate of N_2_O production or reduction potential. However, at T24 and T48, the estimated N_2_O production potential showed a lower trend in the boosted soil compared to the control (Figure 1A,C), a pattern consistently observed across both soil microcosms dedicated to the GC-MS and MOX sensor analysis. Conversely, the estimated N_2_O reduction potential suggested an increase in boosted microcosms over time (Figure 1B,D), reaching higher levels than in the controls at T24 and T48 for both sampling setups. Overall, these predicted potentials may indicate that the boosted treatment might have functionally shifted the bacterial community toward a higher capacity for complete denitrification (i.e., greater capability to reduce N_2_O to N_2_), thus possibly characterized by an enhanced ability to convert produced N_2_O to N_2_ alongside a reduced potential for N_2_O production in proportion to its potential N_2_O production capacity, as observed in liming treatments where the efficiency of the nitrous oxide reductase enzyme was enhanced [36].

### 3.2. Soil Microcosms Volatilome: MOX Sensors & GC-MS

#### 3.2.1. GC-MS Results

**Figure 2:**
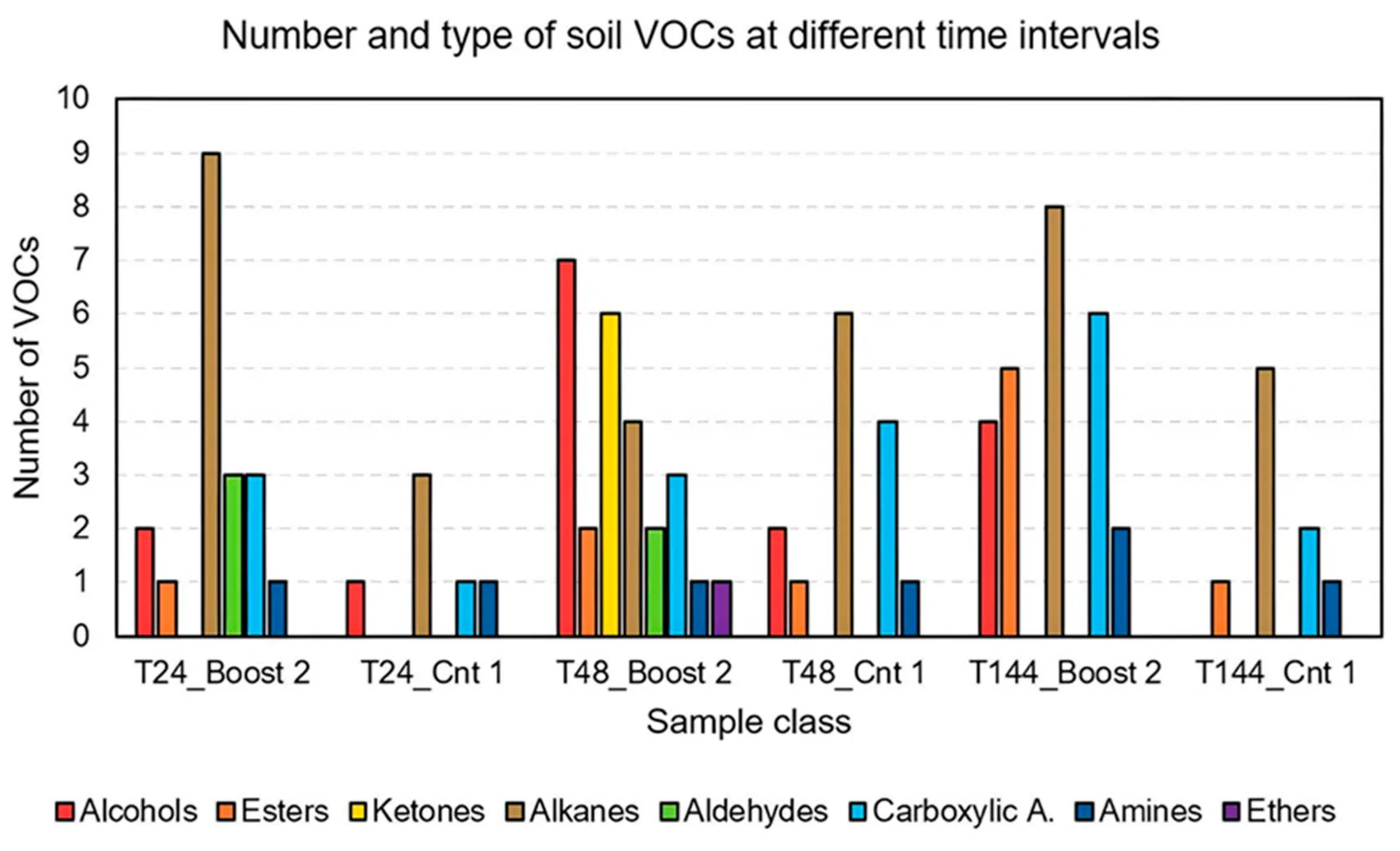
Chemical classification of volatile compounds by GC–MS analysis.

GC–MS analysis revealed clear treatment- and time-dependent differences in the number and distribution of volatile organic compounds among chemical classes in BOOST and control microcosms. As shown in Figure 2, BOOST samples generally exhibited a higher number of detected VOCs and a broader distribution across chemical classes than the corresponding controls. A complete list of the identified compounds and their relative abundances is provided in the Supplementary Materials, Annex S2.

At T24, the BOOST microcosm was characterized mainly by alkanes, together with alcohols, esters, aldehydes, carboxylic acids, and amines, whereas the corresponding control displayed a more limited volatile profile. The largest difference between treatments was observed at T48, when the BOOST condition showed the highest overall number of detected VOCs. This time point was particularly characterized by alcohols, ketones, and alkanes, while ketones and ethers were detected only in the BOOST microcosm. Aldehydes were also observed under BOOST conditions at both T24 and T48.

At T144, differences between treatments remained evident. The BOOST microcosm continued to show a broader volatile profile, with higher numbers of alkanes, carboxylic acids, esters, alcohols, and amines than the corresponding control. Although the distribution of VOCs changed over time, the intermediate sampling phase showed the most pronounced diversification of the volatile profile.

Overall, the GC–MS results indicate that the combined BOOST treatment, comprising increased nitrogen availability, glucose addition, and higher soil water content, was associated with marked temporal changes in the soil volatilome. The observed differences may reflect modifications in microbial activity, substrate availability, oxygen conditions, and other biological or physicochemical processes occurring within the microcosms. These findings should therefore be interpreted as treatment-associated changes in the overall volatile profile rather than as compound-specific evidence of denitrification.

#### 3.2.2. Sensor Signal Behavior and Exploratory Multivariate Analysis

**Figure 3:**
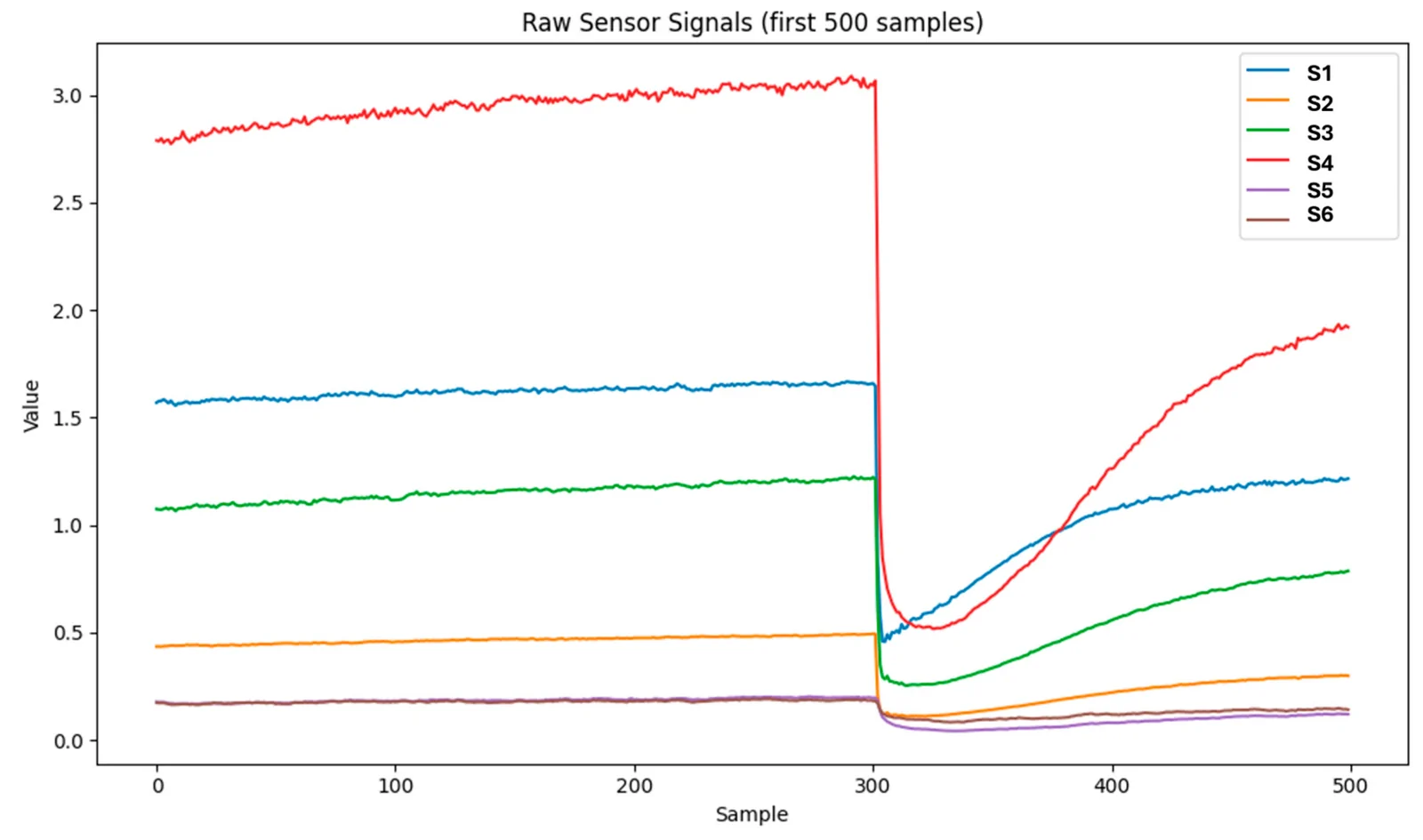
Raw normalized resistance signals (R/R_0_) recorded from the six MOX sensing elements during the first 500 samples of a representative acquisition cycle.

The raw resistance signals were initially examined to characterize the temporal behavior of the six MOX sensing elements before feature extraction and multivariate analysis. As shown in Figure 3, the sensing elements displayed distinct baseline resistance levels and clear changes in response following exposure to the microcosm headspace. Although the magnitude and recovery dynamics differed among sensors, the recorded profiles showed coherent temporal behavior throughout the acquisition cycle, confirming that the array generated measurable and sensor-specific responses under the investigated conditions.

**Figure 4:**
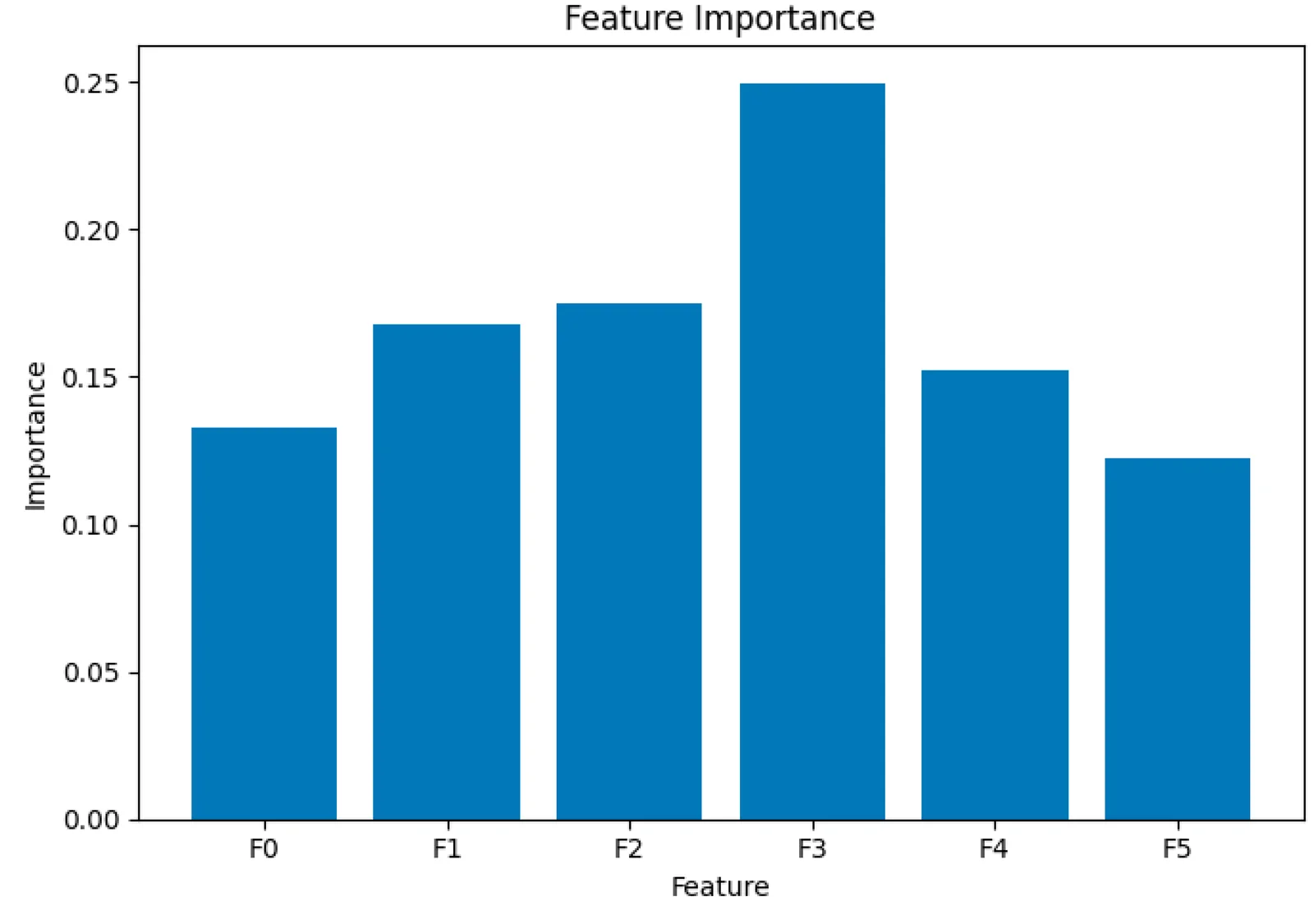
Exploratory Random Forest importance scores of the extracted MOX signal features within the available experimental dataset.

The normalized time-series signals were subsequently converted into numerical descriptors representing different aspects of the sensor response. A Random Forest algorithm was used to obtain an exploratory ranking of these descriptors according to their relative association with the predefined treatment- and time-related acquisition groups within the available dataset. As shown in Figure 4, the extracted features contributed differently to the observed data structure, with F3 displaying the highest relative importance.

Six numerical descriptors were extracted from the normalized resistance time series to characterize the dynamic sensor responses. These included the minimum first derivative (F0), maximum first derivative (F1), area under the response curve (F2), response amplitude (ΔR, F3), signal-to-noise ratio (F4), and overall mean signal value (F5). The area under the response curve was calculated using Simpson’s rule.

These features were subsequently evaluated using a Random Forest algorithm to assess their relative importance in distinguishing the investigated experimental conditions. These importance scores were interpreted exclusively as descriptive indicators of the contribution of each feature within the present dataset and not as estimates of predictive performance or feature generalizability. Because MOX sensors are cross-selective toward complex gaseous mixtures, their responses were interpreted collectively as an overall headspace response pattern rather than as signals attributable to individual compounds. GC–MS analysis therefore provided complementary qualitative information on the chemical changes occurring in the soil volatilome. Overall, the MOX array produced distinct and information-rich resistance profiles suitable for subsequent exploratory multivariate representation by Linear Discriminant Analysis.

#### 3.2.3. MOX Sensors Result

**Figure 5:**
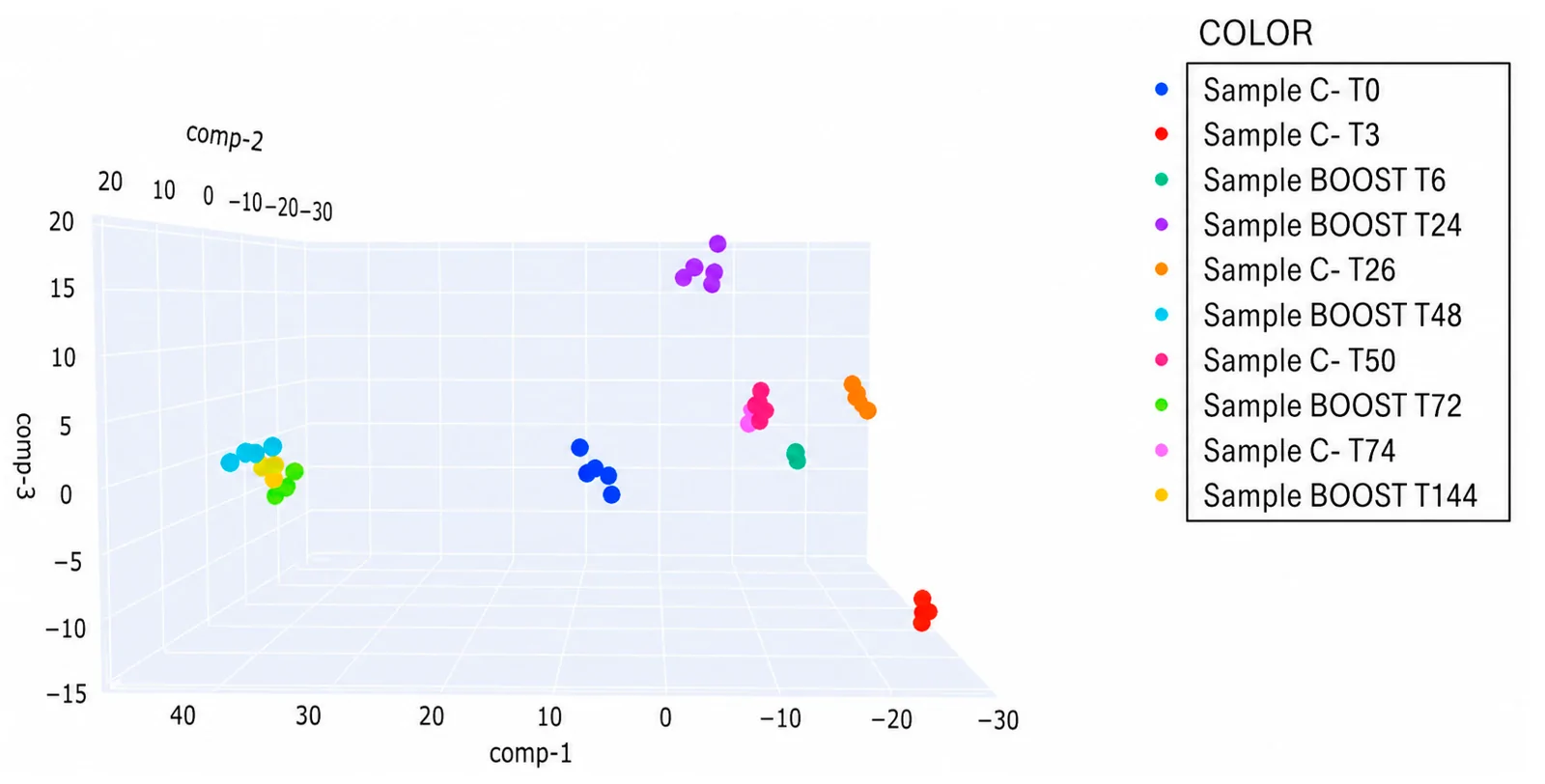
Three-dimensional score plot obtained by multivariate analysis of MOX sensor responses, showing the clustering of samples under control (C) and denitrification-boosted (BOOST) conditions at different time points. Each color represents a specific treatment and sampling time.

Linear Discriminant Analysis was used to represent the multivariate structure of the response patterns generated by the MOX sensor array across the investigated experimental conditions and sampling times. As shown in Figure 5, the technical acquisitions formed distinct clusters within the discriminant space, highlighting systematic differences in the resistance profiles recorded from the individual microcosms. Given the absence of independent biological replication, these clusters should not be interpreted as demonstrating a statistically validated treatment effect. Instead, they describe the internal multivariate structure of the available dataset and indicate that different experimental conditions were associated with distinguishable sensor-response patterns within the investigated microcosms.

Control acquisitions at T0 occupied a specific region of the discriminant space, representative of the initial state of the system. As incubation progressed, both control and BOOST microcosms showed shifts in their respective sensor patterns, indicating a progressive evolution of the gaseous and volatile composition of the headspace. In particular, BOOST acquisitions at approximately 24 h formed a clearly separated cluster from the corresponding control measurements. This phase coincided with the highest N_2_O concentration detected by FTIR. However, this temporal correspondence should not be interpreted as evidence that the MOX array selectively detected N_2_O or that N_2_O was the primary driver of the observed clustering. Because MOX sensors are cross-selective, the response recorded at T24 may reflect the combined effect of multiple concurrent changes in the headspace, including inorganic gases, VOCs, water vapor, and other matrix-related components. The MOX response was therefore interpreted as an integrated fingerprint of the overall headspace composition during this experimental phase, while FTIR and GC–MS provided complementary information on specific gaseous compounds and volatile profiles, respectively. However, this temporal correspondence does not indicate selective detection of N_2_O by the MOX array but rather the ability of the system to capture the overall headspace changes associated with this experimental phase, arising from the combined contribution of volatile and inorganic gases, water vapor, and other components present in the microcosm.

During the intermediate and later stages of the experiment, the MOX acquisitions continued to display treatment- and time-associated distributions, indicating that the array captured changes in the sensor-response patterns over the course of the experiment. The observed patterns were consistent with the changes detected by GC–MS and FTIR, which revealed differences between BOOST and control microcosms in both volatilome composition and target-gas concentrations. This agreement should be interpreted as temporal and analytical complementarity among the three platforms rather than as evidence of compound-specific attribution of the MOX response. FTIR enabled the measurement of selected gaseous compounds, GC–MS characterized the volatile profile, and the MOX array provided a rapidly acquired, integrated response pattern reflecting the overall headspace composition. In this context, the inherent cross-sensitivity of MOX sensors should be considered when interpreting the recorded response patterns, as the sensing elements respond to complex gaseous mixtures rather than to individual compounds. Consequently, the observed resistance changes may reflect the combined influence of VOCs, inorganic gases, humidity, and other headspace components and cannot be unambiguously attributed to a single analyte. For this reason, the MOX response is interpreted as an integrated headspace fingerprint, while compound-specific information is provided by complementary analytical techniques such as FTIR and GC–MS.

A particularly distinct pattern was also observed for the control during the early incubation phase. At this stage, FTIR detected NH_3_ and CH_4_ in the control headspace, whereas these gases were absent or present at lower concentrations in the BOOST microcosms. These compounds, together with VOCs and other headspace constituents, may have contributed to the specific sensor response observed, further supporting the sensitivity of the array to overall variations in the gaseous mixture. Overall, the complementary information provided by FTIR, GC–MS, and MOX sensing contributed to a more comprehensive characterization of the temporal changes in soil headspace composition under the investigated experimental conditions. In particular, the MOX array captured these modifications as distinctive resistance-based response patterns, demonstrating the ability of the system to rapidly and integratively follow the evolution of a complex gaseous matrix under controlled laboratory conditions. Because BOOST and control microcosms also differed in soil water content, part of the recorded variability may have been influenced by headspace humidity. Nevertheless, all measurements were performed at a controlled temperature of 25 °C, thereby reducing variability associated with thermal conditions.

## 4. Conclusions

This study highlights the value of a sequential analytical workflow for characterizing time-dependent changes in soil gas emissions, volatilome composition, and nitrogen-cycling functional markers, while characterizing the response of MOX sensor technology under controlled soil-microcosm conditions. Laboratory-based soil microcosms provided controlled conditions for assessing the effects of the BOOST treatment, which combined mineral nitrogen and glucose addition in saturated soils. BOOST microcosms exhibited marked biochemical and microbial changes, including an early N_2_O emission peak, variations in bacterial abundance and nitrogen-cycling functional gene profiles, and an increased N_2_O reduction potential at later stages. Volatilome analysis revealed a broader volatile profile under BOOST conditions, with time-dependent changes and the emergence of specific VOC classes, reflecting a substantial modification of the soil volatile profile. Complementary MOX measurements generated technically consistent resistance-based response patterns and showed distinct patterns across the investigated experimental conditions and sampling times within the available dataset through exploratory multivariate analysis. Because biological replication was not available for the MOX experiment, these differences cannot yet be interpreted as statistically validated treatment effects. These patterns were interpreted alongside the temporal changes independently observed by FTIR and GC–MS, which provided complementary information on target gases and volatile composition. Overall, the findings indicate that MOX sensors can provide rapid and integrative information on dynamic changes in the overall soil headspace under controlled conditions. The present study therefore provides an exploratory basis for the further characterization and validation of portable, IoT-enabled MOX sensing systems for complex soil-headspace monitoring. Further validation using multiple independently prepared microcosms, direct humidity monitoring, and field-relevant conditions will be required to assess biological reproducibility, predictive performance, and operational applicability. Such systems may ultimately support applications in precision agriculture, environmental monitoring, and sustainable nutrient management.

## Figures and Tables

**Table 1:** Configuration of the MOX sensor array integrated into the S3+ device, showing the two sensor units, each composed of three sensing elements based on undoped and metal-doped SnO_2_ materials.

Sensor Unit	Sensing Element	Active Material	Description
Sensor 1	Element 1	SnO_2_	Undoped
Sensor 1	Element 2	SnO_2_ + Pd	Palladium-doped
Sensor 1	Element 3	SnO_2_ + Au	Gold-doped
Sensor 2	Element 1	SnO_2_	Undoped
Sensor 2	Element 2	SnO_2_ + Pd	Palladium-doped
Sensor 2	Element 3	SnO_2_ + Au	Gold-doped

## Data Availability

The original contributions presented in this study are included in the article. Further inquiries can be directed to the corresponding author.

## Supplementary Materials

The following supporting information can be downloaded at: https://www.mdpi.com/article/10.3390/s26216768/s1, Annex S1: The raw time-series data from the six sensing elements; Annex S2: A complete list of the identified compounds and their relative abundances; Figure S1: Soil laboratory microcosm gasses emissions over 144 hours recorded by the FTIR Gas Analyzer; Figure S2: Quantification of soil (A) total bacteria, (B) nitrogen-fixing bacteria, (C-E) denitrifying bacteria and (F) nitrifying bacteria in control and denitrification-boosted soil laboratory microcosms for GC-MS analysis; Figure S3: Quantification of soil (A) total bacteria, (B) nitrogen-fixing bacteria, (C-E) denitrifying bacteria and (F) nitrifying bacteria in control and denitrification-boosted soil laboratory microcosms for MOX sensor; Table S1: Primer pairs used for qPCR quantification of microbial groups in low and high-field pilots.
