# Supplementary material for: Rapid Assessment of Soil Headspace Dynamics Under Denitrification-Promoting Conditions via an IoT-MOX Sensor Array
Source: Sensors (Basel). 2026 Oct 26;26(21):6768. doi: 10.3390/s26216768 (PMC13765900; doi:10.3390/s26216768)
Supplement: Supplementary file 1 [file sensors-26-06768-s001.zip › Figure S1.pdf]

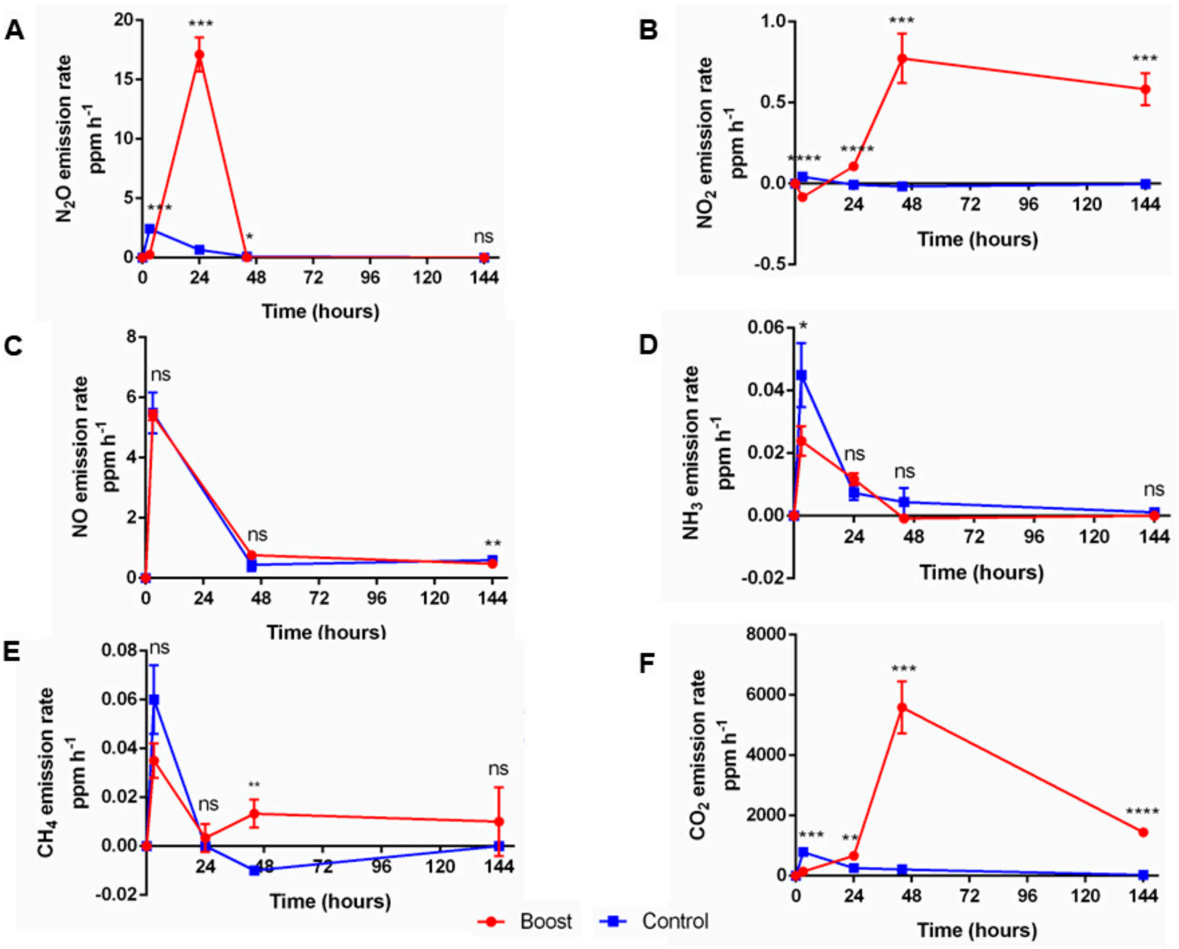

Figure S1. Soil laboratory microcosm gasses emissions over 144 hours recorded by the FTIR Gas Analyzer. Fluxes of N_2O (A), NO_2 (B), NO (C) NH_3 (D) CH_4 and CO_2 expressed in ppm h^{-1} are reported. Bars represent standard error (n = 3). Boosted soil microcosms (red): treated to stimulate denitrification with high mineral N concentration (0.40 g N Kg^{-1} soil), glucose (1 %) and moistened to 100% of field capacity (FC); Control soil microcosms (blue): moistened to 25% of FC. Comparison of gas concentrations (ppm) between Boost and Control treatments for each target gas were evaluated at each time point using Student's t-tests. Statistical variations differences are reported with asterisks (ns, not significant; * P < 0.05; ** P < 0.01, *** P < 0.001, **** P < 0.0001).
