# Supplementary material for: Rapid Assessment of Soil Headspace Dynamics Under Denitrification-Promoting Conditions via an IoT-MOX Sensor Array
Source: Sensors (Basel). 2026 Oct 26;26(21):6768. doi: 10.3390/s26216768 (PMC13765900; doi:10.3390/s26216768)
Supplement: Supplementary file 1 [file sensors-26-06768-s001.zip › Figure S3.pdf]

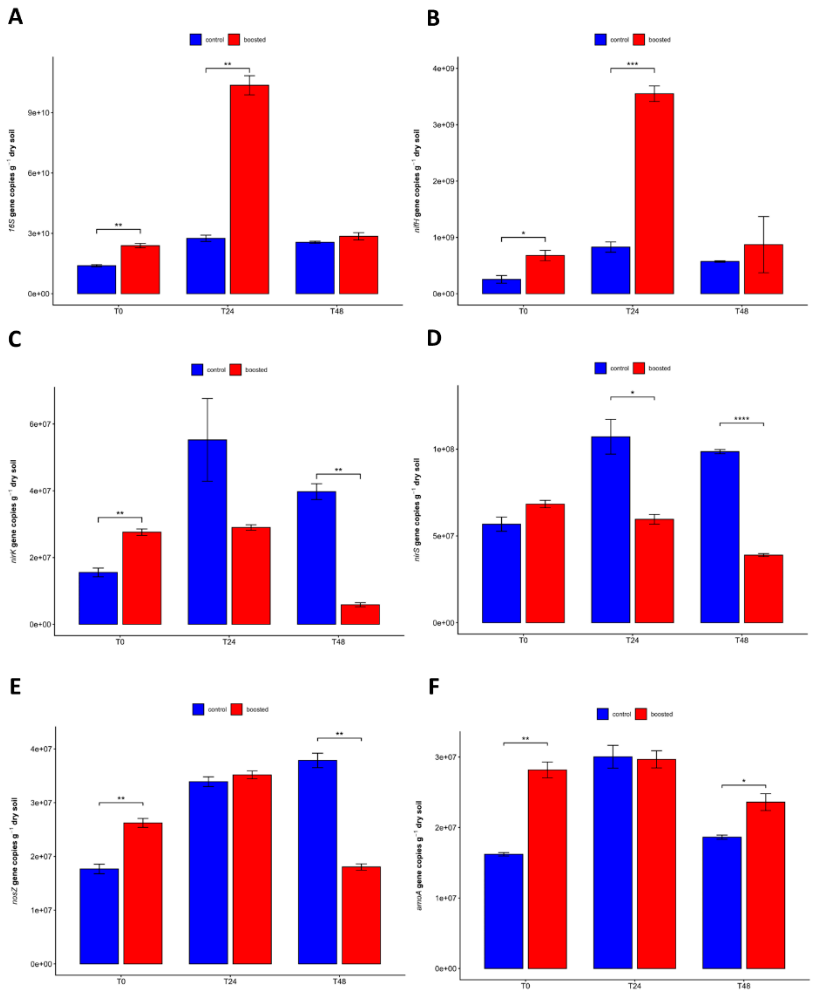

Figure S3. Quantification of soil (A) total bacteria, (B) nitrogen-fixing bacteria, (C-E) denitrifying bacteria and (F) nitrifying bacteria in control and denitrification-boosted soil laboratory microcosms for MOX sensor. The error bars indicate ± the standard errors, whereas the horizontal lines represent mean values. Statistical variations differences are reported with asterisks (ns, not significant; * $P < 0.05$; ** $P < 0.01$, *** $P < 0.001$, **** $P < 0.0001$).
