# Supplementary material for: Rapid Assessment of Soil Headspace Dynamics Under Denitrification-Promoting Conditions via an IoT-MOX Sensor Array
Source: Sensors (Basel). 2026 Oct 26;26(21):6768. doi: 10.3390/s26216768 (PMC13765900; doi:10.3390/s26216768)
Supplement: Supplementary file 1 [file sensors-26-06768-s001.zip › Table S1.pdf]

Table S1. Primer pairs used for qPCR quantification of microbial groups in low and high-field pilots.

Target group	Target gene	Primer sequences	Annealing temperature	Reference
Bacteria	16S rRNA	Bac341F<br>5′-CCTACGGGAGGCAGCAG-3′	60 °C	Muyzer et al., 1993
		Bac805R<br>5′-GGACTACHVGGGTWTCTAA-3′		Caporaso et al., 2011
Nitrogen-fixing bacteria	*nif*H	PolF<br>5′-TGCGAYCCSAARGCBGACTC-3′	55 °C	Poly et al., 2001
		PolR<br>5′-ATSGCCATCATYTCRCCGGA-3′		
Nitrifying bacteria	*amo*A-AOB	AmoA-1F<br>5′-GGGGTTTCTACTGGTGGT-3′	57 °C	Rotthauwe et al., 1997
		AmoA-2R<br>5′-CCCCTCKGSAAAGCCTTCTTC-3′		
Denitrifying bacteria	*nir*K	F1ACu<br>5′-ATCATGGTSCTGCCGCG-3′	57 °C	Throbäck et al., 2004
		R3Cu<br>5′-GCCTCGATCAGRTTGTGGTT-3′		
	*nos*Z	nosZF<br>5′-CGYTGTTCHTCGACAGCCAG-3′	55 °C Scala et al 1998	
		nosZ1733R<br>5′-ATRTCGATCARCTGBTCGTT-3′		
	*nir*S	Cd3aF1<br>5’-GTSAACGTSAAGGARACSGG-3’	52 °C	Throbäck et al., 2004
		R3cd<br>5’-GASTTCGGRTGSGTCTTGA-3’		
